# Improving Visual Field Testing Reliability by Using an Educational Patient Leaflet

**DOI:** 10.7759/cureus.98033

**Published:** 2025-11-28

**Authors:** Basil Suresh, Sam Kathiramalai, Harikesh Kaneshayogan

**Affiliations:** 1 Medicine and Surgery, West Hertfordshire Teaching Hospitals NHS Trust, Watford, GBR; 2 Ophthalmology, West Hertfordshire Teaching Hospitals NHS Trust, Watford, GBR

**Keywords:** glaucoma clinic, glaucoma diagnosis and management, humphrey analyzer, humphrey visual field perimetry, patient understanding, quality improvement study, standard automated perimetry, visual field loss, visual field screening, visual field testing

## Abstract

Introduction

Reliability indices such as fixation losses (FL), false positives (FP), and false negatives (FN) determine the clinical utility of standard automated perimetry (SAP). Patient education can improve test performance, but low-cost, scalable approaches are needed. Our study aimed to evaluate whether a one-page patient leaflet delivered immediately before testing improves Humphrey Swedish Interactive Thresholding Algorithm (SITA) Fast 24-2 reliability.

Methods

We conducted a single-center, non-randomized quality-improvement study in glaucoma follow-up clinics. A total of 39 patients were included in this study. Each eye’s most recent pre-leaflet SAP test was paired with its first post-leaflet SAP test under identical parameters (Humphrey Field Analyzer, SITA Fast 24-2, size III, 31.5 asb). The primary endpoint was composite “full reliability” (FL ≤10%, FP ≤10%, FN ≤10%) per eye, analyzed with McNemar's test and effects reported as the difference in paired proportions with 95% CI. Family-wise error across eyes was controlled by Holm-Bonferroni. The secondary endpoints were continuous changes in FL%, FP%, and FN% per eye, analyzed using the Wilcoxon signed-rank test with Hodges-Lehmann (HL) median change. Analyses were performed per eye without across-eye pooling, and only pairwise complete cases were used. This project was registered locally as a quality-improvement study and adhered to the principles outlined in the Declaration of Helsinki.

Results

We analyzed 39 right and 37 left eyes (patient mean age: 66±15.5 years; mean inter-test interval 6.3 months). For our primary outcome of full reliability, the proportion of reliable tests increased from 31% to 54% in the right eye and from 41% to 65% in the left eye, with both remaining significant after Holm-Bonferroni. For our secondary outcome, FN% decreased significantly (right -4.5 pp and left -4.0 pp), while FL% declined in the right eye (-16.4 pp) with no significant change in the left. FP% showed small, non-significant increases.

Conclusions

A brief, one-page leaflet delivered immediately before SITA Fast testing increased the proportion of fully reliable fields and reduced false-negative responses in routine practice. This low-cost intervention is straightforward to implement and may reduce repeat testing. Confirmation under SITA Faster with a randomized design using visit-level endpoints is warranted.

## Introduction

Standard automated perimetry (SAP) remains a cornerstone in the diagnosis and monitoring of glaucomatous visual field (VF) loss. Among the available perimetric strategies, the Swedish Interactive Threshold Algorithm (SITA) Fast protocol used in the Humphrey field analyzer provides a time-efficient means of assessing visual function while maintaining acceptable test-retest reliability [1,2]. However, the clinical value of SAP critically depends on the reliability of each examination, which may be limited by patient-related factors such as poor understanding, inattention, fatigue, or anxiety during testing [3]. Unreliable VF results can lead to inappropriate clinical decisions, including misclassification of disease severity or erroneous progression analysis [4]. Reliability indices, including fixation losses (FL), false positives (FP), and false negatives (FN), are routinely reported to guide the clinician’s interpretation of a given test. High FL or FP rates typically reflect suboptimal fixation stability or excessive trigger-happy responses, respectively, while elevated FN rates may indicate fatigue or fluctuating attention. 

We aimed to determine whether a one-page leaflet could improve the reliability of VF testing in routine glaucoma outpatient clinics. Previous studies have demonstrated that patient education and preparatory instructions can improve comprehension and performance during SAP, yet the format and clarity of these interventions vary widely across centers [5-7]. At our institution, we observed inconsistent reliability indices among patients undergoing SITA Fast testing. This prompted us to carry out a service-evaluation project to determine whether a concise, patient-friendly informational leaflet could enhance test reliability. The leaflet was designed to explain the purpose of perimetry, the testing process, and key behavioral instructions in plain language. We compared the reliability indices of VF tests performed before and after leaflet introduction, analyzing changes in FL, FP, and FN. By quantifying changes in these indices, we could conclude whether a simple educational intervention can produce a measurable improvement in VF reliability within a real-world clinical setting where audiovisual resources may be impractical.

This work was presented as an academic poster at the UK and Éire Glaucoma Society 2025 annual conference.

## Materials and methods

We conducted a single-center, pre-post quality-improvement study within the ophthalmology service at St Albans City Hospital, a district general hospital in the UK. The study was conducted between 15 July 2025 and 15 October 2025. The intervention was an educational, patient-focused leaflet given immediately before SAP. We then compared each eye’s most recent pre-leaflet field (“baseline”) with its first post-leaflet field (“post-intervention”). A total of 39 patients were included. Inclusion criteria included adults attending glaucoma follow-up with suspected or confirmed primary open-angle glaucoma who underwent SITA Fast 24-2 within the prior 12 months and returned for a post-leaflet SITA Fast 24-2 using identical stimulus parameters. Exclusion criteria included intercurrent ocular surgery or acute ocular events between tests, inability to engage with the leaflet, missing or invalid reliability indices, or any change in perimetry program or strategy. We used the electronic patient records system to collect retrospective data from SAP printouts of previous tests and prospectively collected the same data points from the new post-leaflet test printouts.

**Figure 1:**
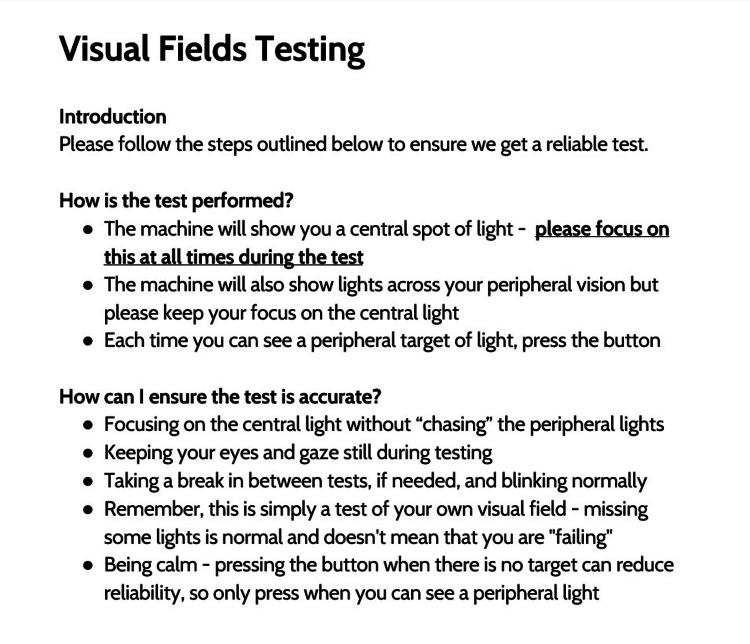
Patient information leaflet

The one-page leaflet (Figure 1 of Appendices) explained how the test is performed, the importance of steady fixation, pressing the response button only when a light is seen, blinking freely, taking breaks between tests, and that missed lights are expected. It aimed to help patients understand the subjective nature of the test and the importance of reliable results while reassuring them to take their time and maintain fixation without visually chasing targets. The leaflet demonstrated high readability, achieving a Flesch Reading Ease score of 77.6 and a Flesch-Kincaid grade of 4.5, consistent with a UK Year 5/US Grade 4-5 reading level, making it suitable for routine patient use. Patients read the leaflet immediately before testing and could ask the technician questions. All tests (pre- and post-leaflet) were carried out by experienced technicians using the Humphrey Field Analyzer (Carl Zeiss Meditec, Dublin, CA, USA) with the following program settings: SITA Fast 24-2, Goldmann size III, white-on-white, background 31.5 asb; fixation-monitoring mode was unchanged within each eye’s pre-post pair. FLs were measured with Heijl-Krakau blind-spot catch trials alongside gaze tracking.

Our primary outcome was a composite binary endpoint for each eye: reliable or unreliable. We considered a test fully reliable when all three indices scored ≤10%. This serves as a conservative, clinically oriented standard, as modest FP/FN rates can materially bias thresholds and global indices, while FL itself remains an imperfect marker of fixation. Therefore, a stringent composite prioritizes dependable fields for accurate monitoring of disease progression. Pre-post change was tested with McNemar’s test (exact p). We report discordant counts (improved vs. worsened), the difference in paired proportions in percentage points (pp) with 95% CI derived from the discordant pairs, and the McNemar matched-pairs odds ratio with Wald 95% CI. Family-wise error across eyes for the primary endpoint was controlled using Holm-Bonferroni. Our secondary endpoints were continuous changes in FL%, FP%, and FN%. Paired pre-post differences were analyzed per eye.

Normality of data was assessed with Shapiro-Wilk and visual inspection (histograms and Q-Q plots). Non-parametric analysis was performed using Wilcoxon signed-rank tests, which were reported alongside the Hodges-Lehmann (HL) median paired change with values for 95% CI, Z, p, and effect size (r=|Z|/√N). Values extracted from perimetry printouts were analyzed as proportions (0-1) in SPSS. Pre- and post-summaries are reported as percentages (%), and paired changes as percentage points (pp; post-pre, negative=improvement). Pairwise missingness (e.g., absent blind-spot detection for FL) was handled by limiting analysis to complete-case pairs per comparison, with N shown for each result. 

Differences were analyzed using IBM SPSS Statistics software. Data were analyzed separately for the right eye and left eye and are reported in parallel. Because outcomes from both eyes of the same participant are correlated, we did not pool across eyes and did not compute a single combined inferential test. IRB approval was not required, as this work was registered as a clinical audit and service evaluation with the local clinical governance unit. Individual consent was waived because only routine care processes were evaluated, and all patient data were de-identified before analysis. This study adhered to the principles outlined in the Declaration of Helsinki. 

## Results

We analyzed the available pre- and post-intervention data from a total of 39 right and 37 left eyes. The mean patient age was 66±15.5 years, with 23 male and 16 female patients. Average pre-leaflet mean deviation (MD) was -3.68 dB (left) and -3.95 dB (right); post-leaflet values were -4.04 dB (left) and -3.13 dB (right). Average pre-leaflet pattern standard deviation (PSD) was 4.69 dB (left) and 4.67 dB (right); post-leaflet values were 5.12 dB (left) and 4.83 dB (right), with the mean follow-up interval being 6.3 months.

Following the leaflet introduction, our primary outcome, the proportion of fully reliable visual field tests, increased significantly in both eyes. In the right eye (n=39), reliability rose from 31% (12/39) pre-leaflet to 54% (21/39) post-leaflet. Eleven eyes improved, and two worsened, with the paired difference being +23.1 pp (95% CI +6.5 to +39.7) and the matched-pairs odds ratio being 5.50 (95% CI 1.22-24.81; exact McNemar p=0.022). In the left eye (n=37), reliability increased from 41% (15/37) to 65% (24/37). Twelve eyes improved and three worsened, with the paired difference being +24.3 pp (95% CI +5.4 to +43.3) and the odds ratio being 4.00 (95% CI 1.13-14.17; p=0.035). Both effects remained significant after Holm-Bonferroni adjustment for the two eyes. For our secondary outcomes (see Table 1 for full outcomes), FN fell significantly in both eyes: right eye -4.5 pp (95% CI -8.5 to -1.0; p=0.005) and left eye -4.0 pp (95% CI -7.5 to -1.0; p=0.005). FL also declined in the right eye: -16.4 pp (95% CI -29.0 to -4.6; p=0.001), but showed no significant change in the left eye: +2.1 pp (95% CI -4.8 to +10.0; p=0.438). FP showed small, non-significant changes: right eye +1.5 pp (95% CI 0.0 to +4.0; p=0.119) and left eye +1.0 pp (95% CI 0.0 to +4.5; p=0.065). Overall, the leaflet’s main continuous-effect signal was a reduction in FN%, with an accompanying improvement in FL% in the right eye and minimal change in FP%. 

**Table 1:** Paired changes in secondary reliability outcomes (Wilcoxon signed-rank tests) This table shows the paired change results per eye for each reliability index. Because paired change=(post-leaflet value)-(pre-leaflet value), negative values denote improvement (lower % is better). HL=Hodges-Lehmann median paired change; 95% CI refers to the HL estimate and is expressed in pp. “Z”=Wilcoxon signed-rank standardized statistic; p-values are two-tailed (nominal, with no multiplicity adjustment for secondary outcomes). “r”=the effect size calculated as |Z|/√N, where Z is the Wilcoxon signed-rank standardized statistic and N is the number of non-zero paired differences contributing to Z. “n (pairs)”=the number of paired eyes with both pre- and post-leaflet values for that outcome (pairwise complete cases). FL, fixation losses; FP, false positives; FN, false negatives; OD/OS, right/left eye; pp, percentage points

Index - eye	n (pairs)	HL median change (pp)	95% CI (pp)	Z-value	p-value	r (effect size)
FL-OD	35	-16.4	-29.0 to -4.6	-3.27	0.001	0.55
FL-OS	32	+2.1	-4.8 to +10.0	-0.78	0.438	0.14
FP-OD	39	+1.5	0.0 to +4.0	-1.56	0.119	0.25
FP-OS	37	+1.0	0.0 to +4.5	-1.85	0.065	0.30
FN-OD	39	-4.5	-8.5 to -1.0	-2.83	0.005	0.45
FN-OS	37	-4.0	-7.5 to -1.0	-2.82	0.005	0.46

Across both categorical and continuous metrics, the leaflet intervention produced measurable improvements in visual field reliability. Gains were driven primarily by reductions in FN responses, suggesting better sustained attention and patient understanding of the task.

## Discussion

Optimizing the reliability of perimetry is essential, as unreliable indices have been shown to have varying effects on both MD and PSD, which can complicate the analysis of glaucoma progression and severity classification [8]. Our study shows that a brief patient-education leaflet delivered immediately before SAP produced a substantial shift in reliability. The proportion of fully reliable fields (all indices ≤10%) increased by approximately 23 pp in both eyes, driven primarily by reductions in FN. As FNs can reflect lapses of attention and fatigue, this pattern suggests improved task understanding, pacing, and anxiety management rather than just “trigger discipline.” These results align with previous research in this area, demonstrating the clear benefits of patient education on test reliability. No studies have examined the effect of patient-education leaflets on SAP reliability; however, they have instead assessed the impact of audiovisual interventions [5,6,9]. Patient education remains a vital component for high-quality investigation and monitoring of disease, not just in perimetry but also in the wider medical field [10,11]. Though less frequently considered compared to FL or FP, reducing FN rates is vital, as even low rates of FN have been associated with cases of incorrect glaucoma diagnosis in patients who have disc cupping within the normal physiologic range [12]. It should be remembered, however, that FN rates tend to be higher in eyes with significant field loss, making it a poor indicator of test reliability in such patient cases [13].

FP responses in SAP can artificially elevate point sensitivities and MD, creating deceptively clean fields that can mask true defects and distort indices [14]. Therefore, falsely high FP rates render tests unreliable, with the impact most pronounced in advanced glaucoma. This index is known to have the largest impact on overall test reliability in SAP, with the extent of this impact varying depending on the severity of glaucoma [15]. Our results for FP showed only small, non-significant changes, though pre-leaflet, approximately 80% of tests already met FP≤10%, creating a ceiling effect that likely limited detectable post-leaflet change. FL events (Heijl-Krakau blind-spot “seen” events) flag potential misfixation or blind-spot mapping errors. Elevated rates, considered to be approximately >20% in general literature, typically indicate an unreliable Humphrey field and should be weighed alongside the gaze-tracker trace [16]. Our results show that FL improved in the right eye but not the left, which may be due to factors such as inattention or fatigue, as testing was generally performed with the right eye first and without randomization of eye order. A study by Kelly and associates indicated that testing order and fatigue may have small but statistically significant perimetric fatigue effects on the second eye [17]. Prior studies have also established methodological limitations of blind-spot catch trials, highlighting them to be a somewhat poor proxy for fixation with gaze tracking [18]. This is an additional reason why our composite threshold of ≤10% per index is intentionally stringent. While many services tolerate higher cutoffs, a stricter service standard prioritizes dependable fields for accurately establishing baseline field status and progression analysis.

Methodologically, analyzing paired eyes separately without across-eye pooling respects within-patient correlation and clinical reality (clinicians act on eye-level reliability). Using nonparametric paired estimators (HL median change with CIs) and exact McNemar tests provides robust inference for skewed indices, and family-wise error was controlled across eyes. Using simple educational aids to minimize unreliable fields also benefits service operation, aside from low-cost implementation, such as fewer same-day repeats, smoother clinic flow, and increased confidence in longitudinal change detection, especially in moderate-advanced VF loss, where variability is highest. The behavioral principles reinforced in our leaflet (maintaining steady fixation, responding only to seen stimuli, blinking normally, and understanding that missed lights are expected) remain applicable as services transition to SITA Faster. However, future re-evaluations are necessary and should incorporate SITA Faster-specific reliability indicators, as it does not measure false-negative or blind-spot fixation-loss percentages and instead relies more heavily on enhanced gaze-tracking traces and response-time modeling [19]. These differences mean that reliability must be assessed using the metrics appropriate to the newer strategy.

The main limitations of our study include its single-center focus, meaning that findings may reflect local patient mix, technician practices, and workflows, somewhat limiting external validity and generalizability to other settings. Our sample size also limits precision for small effects and precluded multivariable or clustered model analysis. We did, however, prioritize paired, eye-level analyses with absolute risk differences and HL median changes, which are clinically interpretable. SAP outcomes are also prone to learning effects, with patients having improved performance with repeated exposure, particularly at initial visits. Accordingly, some of the post-leaflet gains may reflect practice rather than the intervention [20]. Although within-eye pairing reduces between-patient confounding, a one-group pre-post design remains susceptible to regression to the mean and other time-varying confounders in the absence of a concurrent control. Another point to consider is that the right eye is usually tested first by convention, with many perimetry setups defaulting to the right eye first, which can bias first vs. second-eye performance. It is important to acknowledge that disease severity may also influence FN, and although within-eye pairing mitigates this, it does not eliminate it completely. Our primary composite outcome is also constrained by FL, which is an imperfect proxy for fixation. The stringent ≤10% threshold for all three indices means that FL can veto reliability even when FP and FN are acceptable. Therefore, given FL’s limitations, clinicians are always advised to interpret FL with caution alongside FP and FN values.

## Conclusions

A simple, one-page leaflet delivered immediately before testing produced a significant improvement in Humphrey SITA Fast reliability. This was largely driven by reductions in FN and a concurrent improvement in FL in the right eye. As the intervention is low-cost, quick to administer, and easily embedded into existing workflows, it is well-suited to routine glaucoma outpatient follow-up settings and may reduce repeat testing and streamline clinics. Findings should be interpreted alongside our design constraints (single center, non-randomized before-and-after design), but the magnitude and consistency of effect support pragmatic adoption while further evidence accrues.

Next steps include confirmation under SITA Faster, incorporation of gaze-based and response-time metrics, and randomized evaluations with health-economic endpoints (cost per reliable test, time saved, and repeat-test rates). Future work may also test durability over time, technician-to-technician variability, and performance in key subgroups (age, baseline field loss, and language or cognitive barriers), with translated materials to maximize equity and generalizability. Taken together, our results indicate that targeted, pre-test education is a practical lever to enhance perimetric data quality, which may improve the accuracy of long-term glaucoma monitoring.
